# Les déterminants du climat émotionnel dans les familles de patients avec schizophrénie: étude transversale sur 50 familles

**DOI:** 10.11604/pamj.2023.44.11.18919

**Published:** 2023-01-05

**Authors:** Amine Bout, Nabil Berhili, Chadya Aarab, Rachid Aalouane

**Affiliations:** 1 Centre Hospitalier Hassan II de Fès, Fès, Maroc

**Keywords:** Emotion exprimée, schizophrénie, famille, Expressed emotion, schizophrenia, family

## Abstract

**Introduction:**

la notion d´émotion exprimée (EE) remonte aux années soixante et désigne l´attitude de la famille envers un proche schizophrène. Elle se décline en trois éléments qui sont les commentaires critiques, l´hostilité et le surinvestissement émotionnel. Une riche littérature prouve qu´un haut niveau d´EE est un facteur de rechute de la schizophrénie. Notre étude a eu comme objectif de mesurer, sur un échantillon marocain de patients avec schizophrénie, le niveau d´EE dans leur milieu familial, puis dans un deuxième temps de chercher les facteurs associés à un haut niveau d´EE.

**Méthodes:**

cinquante (50) patients schizophrènes stables avec chacun un proche impliqué dans les soins sont recrutés en marge de la consultation. Des éléments sociodémographiques sont recueillis et l´échelle The Family Attitude Scale (FAS) est passée au proche. Sont également recueillies des données concernant les représentations mentales des proches sur le malade et la maladie. L´analyse statistique a été réalisée par le logiciel SPSS et s´est basée sur des tests chi 2 ainsi que des tests T pour échantillons indépendants.

**Résultats:**

quarante-huit pourcent (48%) des proches avaient un haut niveau d´EE. Le haut niveau EE était associé à la présence d´un sentiment de honte vis-à-vis du proche malade. Il est également associé à la dépendance au cannabis. Un bas niveau d´EE est associé au fait que le patient prenne en charge sa famille financièrement.

**Conclusion:**

la connaissance des déterminants d´un haut niveau EE dans notre contexte socio-culturel est essentielle afin de diriger toute intervention de psychoéducation visant à réduire ce niveau d´EE.

English abstract

## Abstract (en)

**Introduction:**

expressed emotion (EE) is a concept which dates back to the sixties and refers to the attitude that relatives have toward a family member with schizophrenia. It comprises three behavioral patterns: criticism, hostility, and emotional overinvolvement. An important body of literature has shown that high expressed emotion (EE) is a factor of relapse in schizophrenia. The purpose of our study was to measure expressed emotion in the families of a Moroccan sample of patients and then to investigate factors associated with high EE.

**Methods:**

fifty (50) patients with stable schizophrenia, each with a relative involved in their care, were recruited during outpatients visits. Sociodemographic data were collected and the FAS scale was used by relatives. Data were also collected from the mental representations of relatives about the patient and disease. Statistical analysis was carried out using the SPSS software and was based on the Chi 2 tests as well as T tests for independent samples.

**Results:**

forty-eight percent (48%) of relatives had a high EE. High EE was associated with a feeling of shame toward the patient. It was also associated with cannabis addiction. Low EE was associated with the fact that the patient financially took care of his family.

**Conclusion:**

the knowledge of the determinants of high EE in our socio-cultural context is essential in order to direct any psycho-educational intervention aimed at reducing EE.

## Introduction

Brown crée en 1960 la notion d´émotion exprimée (EE) qui reflète le climat affectif familial et désigne l´ensemble des attitudes des proches vis-à-vis d´un malade. Elle se décline en trois dimensions essentielles à savoir: les commentaires critiques (CC), le surinvestissement émotionnel et l´hostilité [1]. L´auteur a noté que les patients qui obtenaient leur congé de l'hôpital pour retourner dans une ambiance familiale très chargée émotivement rechutaient plus que ceux qui retournaient vivre dans un environnement où l'émotivité était plus tempérée. Il a mis au point l'entrevue *Camberwell Family Interview (CFI)*.

Le *CFI* est administré à un proche en privé et est enregistré sur bande vidéo. L'examinateur interroge le proche sur différents aspects de la vie familiale, y compris l'histoire de la maladie, les symptômes et comportements spécifiques, les tâches ménagères et la qualité des liens affectifs entre le patient et ses proches. À l'origine, l'entrevue durait quatre heures. Sa durée a été ramenée à 90 minutes par Vaughn et Leff [2]. La première étude à avoir utilisé le *CFI*, publiée en 1972, a démontré la prédictibilité de la rechute à 9 mois [3]. Cette méthode a été reprise par Vaughn et Leff [2]. Hooley a combiné les résultats de ces deux études, qui étaient assez semblables, et en a conclu que si 13% des patients provenant de foyers à EE faible rechutaient au cours de la période de suivi de neuf mois, cette proportion passait à 51% chez ceux qui provenaient de foyers cotés EE élevée [4].

Depuis la publication de ces études en Angleterre, la méthodologie de base a été reproduite dans divers pays et cultures à travers le monde et elles ont contribué à confirmer que le niveau d´EE élevée est un facteur prédictif de la rechute[5-11]. Une méta-analyse a regroupé 12 études de ce type et a permis de démontrer que le risque de rechute au cours des neuf mois suivant la sortie de l´hôpital est d´environ 3,7 fois plus grand pour un patient schizophrène vivant dans un foyer coté EE élevée que pour un patient vivant dans une ambiance familiale cotée EE faible [12]. Malgré une validité et une reproductibilité impressionnantes de ces résultats, la portée réelle de l'émotion exprimée et les mécanismes qui l´associent à la rechute, ainsi que le sens de cette association restent mal élucidés. Les études réalisées ultérieurement se sont attachées à identifier les caractéristiques des patients associées à une EE familiale élevée. Ont été explorés des facteurs sociodémographiques et culturels ainsi que des facteurs liés à la maladie et à son évolution [13-17]. Il en demeure que la littérature sur les déterminants du niveau d´EE reste disparate et peu concluante. En matière d´outils de mesure, *CFI* constitue le Gold-standard mais son utilisation est limitée par son temps de passation et de cotation qui peut atteindre 5 heures. D´autres outils psychométriques plus pratiques ont été créés. A savoir, *The Five Minute Speech Sample (FMSS)* crée par Magana en 1986 et qui fut validé contre le CFI [18] et *The Family Attitude Scale (FAS)* qui est un auto-questionnaire à 30 *Items* proposé par Kavanagh *et al*. [19] qui rapportent une grande consistance interne et une bonne validité sur une population de parents d´étudiants. Kavanagh [20] a aussi retrouvé une bonne validité chez les parents et les proches de schizophrènes avec notamment une bonne corrélation avec les CC et l´hostilité mesurés par la CFI.

Notre travail s´est proposé de mesurer le niveau des EE dans une population marocaine de proches de patients schizophrènes en rémission. L´objectif principal est de mesurer la moyenne du score d´EE et de dégager le pourcentage de familles étiquetées avec un haut niveau d´EE. Nous avions également comme objectif l´étude des facteurs liés à un haut niveau d´EE en tenant compte de la composante culturelle locale. Enfin, nous avons cherché à vérifier une hypothèse basée sur une observation clinique concernant le rôle que peuvent jouer les représentations mentales des proches sur la maladie dans le déclanchement d´une attitude critique ou hostile, notamment le caractère perçu comme volontaire des troubles de comportement et la responsabilité du malade comme déclencheur de son trouble. Nous vérifions également le rôle que peut jouer le sentiment de honte dans l´attitude du proche.

## Méthodes

Nous avons recruté 50 patients avec un diagnostic de schizophrénie selon les critères DSM-IVR (Diagnostic et Statistique des troubles mentaux), ainsi qu´un membre de leurs proches présents à la consultation externe du centre de diagnostic du service de psychiatrie au CHU Hassan II Fès (Maroc). Nous avons procédé à un échantillonnage de convenance et le recrutement s´est fait sur les mois d´août et septembre 2014. Nous avons inclus dans notre étude les patients entre 18 et 60 ans, stabilisés depuis plus de 6 mois avec un suivi régulier et une bonne observance thérapeutique. Ont été exclus les patients en rechute, les patients avec trouble de l´humeur ou retard mental. Le proche devant être investi dans la prise en charge du patient et vivant sous le même toit. Il s´agit du conjoint ou un parent de premier degré : mère, père, frère ou sœur. Le patient et le proche ont reçu des explications sur l´étude et ont donné leur consentement pour y participer.

Nous avons proposé ensuite un hétéro-questionnaire avec recueil des données sociodémographiques du proche, suivi de questions sur la représentation de la maladie du proche et son vécu par rapport à celle-ci en l´occurrence l´attribution de la maladie au comportement du malade; l´attribution de la maladie à la consommation de drogues; le jugement moral par rapport au comportement du patient et le sentiment de honte vis-à-vis du malade. Le proche à par la suite répondu à l´auto-questionnaire FAS. Nous avons passé l´échelle PANSS (échelle des symptômes positifs et négatifs). Enfin, Les critères DSM IV ont été utilisés pour caractériser l´usage de drogues.

L´étude statistique, réalisée en collaboration avec le service d´épidémiologie du CHU Hassan II de Fès, a consisté tout d´abord en une description de l´échantillon selon les caractéristiques sociodémographiques, les représentations autour de la maladie et les résultats des mesures des EE. Ensuite, la recherche de facteurs associés à un statut haut ou bas du niveau d´EE s´est faite à l´aide de tests chi-deux. Nous avons également pris en compte le caractère continu du score du *FAS* et avons cherché des corrélations entre ce score et les facteurs suspectés d´avoir un lien avec le niveau d´EE, ceci a été réalisé à l´aide de tests T pour échantillons indépendants.

## Résultats

La moyenne d´âge des patients est de 34 ans avec un écart type de 8,6 avec 84% d´hommes et 16% de femmes. Quatre-vingt-deux pour cent (82%) des malades sont célibataires, 14% sont mariés et 4% sont divorcés. Seulement 12% ont un travail régulier. Le niveau scolaire des patients se répartit comme suit: 18% d´analphabètes; 44% ont atteint le primaire, 34% ont un niveau secondaire et 4% ont un niveau universitaire. Vingt-huit pour cent des patients participent financièrement dans leur ménage et 10% sont en charge de la famille. Les formes cliniques selon le DSM IV sont: schizophrénie paranoïde (66%); schizophrénie désorganisée (30%); schizophrénie indifférenciée (2%); schizophrénie catatonique (2%). Le traitement antipsychotique est à base de classiques dans 72% des cas et d´atypiques pour 28%. L´usage de cannabis concerne 34% des participants avec 10% d´abus de cannabis et 24% de patients dépendants au cannabis. L´ancienneté de la maladie varie de 1 à 20 ans avec une moyenne de 7,4 années et un écart type de 4,879. La durée moyenne cumulée d´hospitalisation est de 26 jours avec un écart type de 36 jours.

L´âge moyen des proches est de 55 +/-12. Le lien de parenté est réparti comme suit: 46% de mères; 26% de pères; 20% de frères ou sœurs 6% de conjoints et 1 fils. Avec 56% d´analphabétisme. Dans 74% des cas il s´agit de familles nucléaires. Les familles ont un revenu moyen (entre 3000 et 6000 MAD) dans 38% des cas et bas dans le reste de l´échantillon à savoir 62%. Les deux tiers des familles sont urbaines. Seulement 20% des proches sont au courant du diagnostic de leur proche malade. Trente-huit pour cent (38%) pensent que le malade est responsable de son trouble. Quarante pour cent (40%) des proches pensent que le patient agit de manière immorale. Enfin, 56% des proches éprouvent un sentiment de honte vis-à-vis du patient et de sa maladie. La moyenne du score du *FAS* est de 54,1 avec un écart type de 26,14. Pour un cutoff de 50 à l´échelle du *FAS* 48% des ménages affichent un haut niveau d´émotion exprimée. Les patients qui sont en charge de leurs familles ont montré une association significative avec un bas niveau d´EE, p=0,019. Un test chi-deux croisant l´usage de cannabis avec le statut EE ne trouve pas d´association significative entre l´usage et le haut niveau EE avec p=0,081. Tandis que le test chi-deux comparant les statuts EE chez les patients dépendants versus non dépendants au cannabis trouve une association significative avec p=0,045. Les tests T comparant le score du *FAS* entre consommateurs de cannabis et non consommateurs trouve une différence significative avec p=0,011. La comparaison du score entre patients dépendants et non dépendants trouve une différence très significative dans les scores du *FAS* avec p=0,004.

Aucune des représentations mentales sur la maladie n´affecte le niveau d´EE, par contre les proches qui disent exprimer un sentiment de honte vis-à-vis du proche malade ont une association fortement significative avec un haut niveau d´EE (p=0,008). Un test khi-deux retrouve une association entre le jugement moral et le sentiment de honte (p=0,001). On a retrouvé également une association entre le sentiment de honte chez les proches et le fait qu´ils pensent que le patient est responsable de sa maladie (p=0,002). A l´aide d´un test T pour échantillons indépendants nous avons comparé les moyennes des trois scores du PANSS selon si le niveau d´EE est bas ou haut. Aucune association n´a été retrouvée. Une analyse bivariée a été effectuée à la recherche d´associations ente le score du FAS et les scores respectifs du PANSS, et aucune corrélation n´a été retrouvée. Le Tableau 1 regroupe les résultats sur les déterminants du niveau d´émotion exprimée.

**Tableau 1:** les déterminants du climat émotionnel dans les ménages des schizophrènes

			FAS (score)		P	Statut EE		P
			Moyenne	Ecart type		Haut Niveau	Bas niveau	
Sexe	M	42	54,14	26,48	0,38	19	23	0,45
	F	8	53,88	25,98		5	3	
Activité professionnelle stable	sans	44	55,95	26,178	0,43	22	22	0,66
	avec	6	40,50	23,510		2	4	
Traitement antipsychotique	Classique	36	53,44	25,722	0,779	17	19	1,00
	atypique	14	55,79	28,121		7	7	
Connaissance de la maladie	Oui	10	43,80	29,984	0,166	3	7	0,294
	Non	40	56,68	24,84		21	19	
Attribution de la maladie à une drogue	Oui	35	52,31	25,731	0,466	16	19	0,760
	Non	15	58,27	27,528		8	7	
Attribution de la maladie au malade	Oui	19	56,32	28,783	0,644	10	9	0,772
	Non	31	52,74	24,786		14	17	
Jugement immoral	Oui	20	58,55	28,369	0,323	10	10	1,00
	Non	30	51,13	24,594		14	16	
Sentiment de honte	Oui	28	62,57	25,703	0,008	18	10	**0,012**
	Non	22	43,32	22,979		6	16	
Famille nucléaire	Oui	37	57,03	27,178	0,184	20	17	0,202
	Non	13	45,77	21,761		4	9	
Participation financière dans le ménage	Oui	14	43,71	22,909	0,08	5	9	0,352
	Non	36	58,14	26,497		19	17	
Le patient prend en charge la famille	Oui	6	31,00	12,570	0,019	0	6	0,023
	Non	44	57,25	25,998		24	20	
Lieu de résidence	Rural	17	58,59	26,125	0,389	9	8	0,767
	Urbain	33	51,79	26,252		15	18	
Consommation de cannabis	Oui	17	67,00	26,446	0,081	11	6	0,011
	Non	33	47,45	23,728		13	20	
Dépendance au cannabis	Oui	12	72,58	26,134	0,004	9	3	0,047
	Non	38	48,26	23,587		15	23	

## Discussion

Notre travail apporte les toutes premières données sur la notion d´EE au Maroc qui pourtant revêt un poids considérable en clinique quotidienne et impacte directement le cours de la schizophrénie. Ce travail apporte également une mesure du niveau des EE à distance de la rechute afin de minimiser l´impact inverse que peut avoir la rechute schizophrénique et la détresse liée à celle-ci sur la réaction et l´attitude des proches. Nous avons retrouvé un taux de 48% de proches étiquetés avec un haut niveau d´EE. Avec une moyenne de 54,1 +/- 26,14. Sur une population de 50 proches de patient avec comorbidité psychose et usage de substance, Kavanagh [20] retrouve un score de *FAS* moyen de 55 et 20,7 d´écart type avec 77% ayant un haut niveau d´EE. Fujita *et al*. [21] retrouvent 22.0% de haut niveau EE avec une moyenne du FAS de 39,9 +/- 20,4. Nos chiffres s´approchent beaucoup en terme de moyenne de score et son intervalle de confiance des chiffres retrouvés par l´auteur de l´échelle et aussi de l´étude japonaise sus décrite. Les travaux de Kavanagh et Fujita auxquelles nous avons comparé nos résultats avaient aussi pour objectif d´évaluer la sensibilité et la spécificité de l´échelle *FAS* versus l´échelle *CFI* qui est le gold standard en matière de mesure du niveau EE. Kavanagh retrouve une corrélation positive significative entre les deux échelles sur les sous échelles *CFI* des critiques (r=0.44, P=0.001), et d´hostilité (r=0.41, P < 0.01), et aussi mais plus faiblement avec les sous échelles du surinvestissement (r=0.27, P < 0.05). Ce même travail prouve que le *FAS* élevé prédit la rechute dans un échantillon de proches de patients schizophrènes, mais avec une relation moins forte qu´avec le *CFI*. Fujita sur la traduction japonaise retrouve 100% de sensibilité et 85% de spécificité par rapport aux commentaires critiques mesurés par le *CFI*. Le *FAS* reflète aussi l´état psychologique et émotionnel des proches. Ces données sur notre outil de mesure nous permettent de comparer nos résultats sur le niveau d´EE avec une fiabilité acceptable. En effet, nous résumons dans le Tableau 2, la proportion de proches étiquetés EE élevée dans plusieurs études occidentales qui restent comparables à la nôtre en termes de taille et qui s´adresse à des parents de premier degré de patients schizophrènes. Ces travaux trouvent en somme des taux proches voire plus élevés de familles avec un haut niveau d´EE.

**Tableau 2:** niveau d’émotion exprimée dans les études

Etudes	Effectif	Haut niveau EE	Bas niveau EE	%
Brewin *et al*. (1991)	58	43	15	74%
Weisman *et al*. (1993)	46	23	23	50%
Barrowclough *et al*. (1994)	60	41	19	68%
Hooley and Licht (1997)	43	32	11	74%
Weisman, Nuechterlein, Goldstein, and Snyder (1998)	40	29	11	72%
Wendel *et al*. (2000)	52	40	12	76%
Barrowclough, Lobban *et al*. (2001)	20	0	20	0%
Tarrier et al. (2002)	100	48	52	48%

Un certain nombre d´études ont essayé de comprendre le rôle des différences culturelles et leur impact sur l´attitude des proches des malades. Une étude iranienne [22] retrouve 56% de haut niveau EE, 23% et 28% respectivement en Chine [23] et en Inde [24]. Plus proche de notre contexte, un travail égyptien [25] retrouve 55% de haut niveau EE. Il suggère que les patients égyptiens tolèrent mieux les commentaires critiques par rapport aux patients occidentaux. Les mêmes constats furent retrouvés dans une autre étude [26] qui suggère que la proximité avec le patient de par la nature des interactions sociales dans les sociétés traditionnelles pourrait éventuellement être un facteur protecteur et non vécu par le patient comme une intrusion. Sur les déterminants du niveau d´EE: Bensen [14] s´est basé sur 32 études évoquant de possibles facteurs prédictifs d´un haut niveau d´EE puis il a évalué l´impact de 28 variables d´entre-elles, comme la nature du proche, le sexe, le fait que le patient exerce un emploi stable, le niveau économique, le nombre de proches sous le même toit. Il rapporte comme prédicteurs robustes d´un niveau élevé de critique: l´absence d'un emploi rémunéré, plus de trois hospitalisations précédentes, des comportements turbulents rapportés par les parents, et plus encore l´altération du fonctionnement cognitif à l'admission. L´hostilité avait comme prédicteurs robustes l´absence d'emplois et plus de trois hospitalisations précédentes. Dans notre travail, seul le fait que le patient soit la principale personne en charge de la famille était corrélé à un bas niveau EE (p=0,023). Tandis que le fait d´avoir un travail stable n´est pas associé à un haut score du *FAS* ni le fait de participer financièrement dans le ménage. Ceci pourrait être expliqué par le poids de la prise en charge financière du patient, assumé complètement par les proches en l´absence d´aide sociale et de structures adaptées, et qui pourrait être source de détresse sachant que la détresse psychologique est associée à un score élevé du *FAS* [20,21]. Cette relation entre EE et la charge portée par les parents est mise en évidence par Scazufca et Kuipers [17] qui ont montré une forte association entre la baisse de la charge et difficultés liées aux soins et la baisse du niveau d´EE. Par ailleurs nous n´avons retrouvé aucune corrélation entre le nombre d´hospitalisation et le score du *FAS*.

Un des éléments importants dans la relation entre proches et malades, plus encore dans notre contexte, est la consommation de cannabis. Dans notre étude, le score de *FAS* est significativement plus élevé chez les consommateurs de cannabis par rapport aux non consommateurs. De plus, les patients dépendants au cannabis ont un *FAS* plus élevé et le statut haut niveau EE est fortement lié à la consommation de cannabis ce qui nous pousse à formuler l´hypothèse que ce haut niveau EE est secondaire à une attitude critique vis-à-vis de la consommation elle-même pointée comme responsable de la maladie par la famille. L´attribution de la maladie à la consommation de cannabis dans l´imaginaire collectif marocain jouerait un rôle dans cette attitude critique, néanmoins la taille réduite de la sous population ou le patient est consommateur de cannabis n´a pas permis de vérifier l´association entre l´attribution de la maladie au cannabis et le niveau d´EE. Dans la littérature, peu de travaux ont été consacrés à la relation entre EE et usage de cannabis. Pourmand [27] va jusqu´à suggérer que l´effet du cannabis comme facteur de rechute peut se faire à travers un haut niveau EE et non pas un effet psychopharmacologique de la molécule. D´un autre coté une étude récente [28] a relevé une relation longitudinale après un premier épisode de nature psychotique. Elle retrouve qu´une attitude critique au départ de l´étude prédit un mésusage de cannabis tandis que le mésusage de cannabis au départ de l´étude ne prédit pas un haut niveau EE. Selon ce travail la critique pourrait être un facteur stressant et l´usage de cannabis un moyen de gérer ce stress (moyen de coping). Le sens de l´association entre EE et cannabis est complexe et pourrait être donc bidirectionnel. Une meilleure connaissance de l´interaction entre ces deux éléments reconnus comme étant des facteurs de rechutes de la schizophrénie est nécessaire afin de guider les interventions de psychoéducation. Par ailleurs, une étude [29] comparant 13 facteurs potentiellement prédicteurs de rechute schizophréniques retrouve que le haut niveau EE est le facteur de rechute le plus puissant devant la consommation de cannabis.

Nous avons également étudié le lien entre les symptômes résiduels du patient et le niveau d´EE dans sa famille. Aucune corrélation n´a été retrouvée entre les différentes dimensions mesurées par le PANSS et le niveau d´EE. Le même constat est fait par Lopez *et al*. [30] n´ayant pas trouvé non plus de relation entre la sévérité des symptômes mesurés par le PANSS et le niveau d´EE. Bentsen [14] ne retrouve pas non plus de lien entre les dimensions du PANSS et le niveau EE sauf pour les symptômes cognitifs. Cette absence de lien entre la sévérité des différentes dimensions de la maladie et le niveau EE remet relativement en question la critique souvent faite au concept d´EE qui consiste à dire que ce niveau n´est que le reflet immédiat des symptômes du malade. Un résultat important de notre travail est la forte association retrouvée entre le fait d´éprouver un sentiment de honte envers le patient et le niveau élevé d´EE en effet ce facteur est peu étudié jusque-là. Wasserman *et al*. retrouvent cette association chez 72 proches de patients schizophrènes ou il a pu prédire un haut niveau d´EE chez les membres de familles des patients avec un score élevé de sentiment de honte [31]. D´autres travaux ont déjà suggéré cette association dans d´autres types de populations [32-34]. De Mamani [35] et McMurrich [36] ont utilisé une approche dispositionnelle dans l´évaluation du sentiment de honte et n´ont pas objectivé de relation entre EE et honte. Quant à la nature de cette possible association, Tracy et Robins [37]soutiennent que la honte est en corrélation avec une tendance à blâmer les autres en se basant sur des attributions externes comme événement déclenchant la honte. Ces attributions provoquent une attitude critique défensive à l'égard de ceux qui sont impliqués dans la situation qui déclenche la honte ainsi que la colère, la rage, et l'hostilité.

Toujours afin de comprendre la dynamique qui aboutit à un haut ou bas niveau EE, nous avons exploré les représentations mentales des proches et leurs croyances par rapport à la maladie et ses manifestations. Et en se basant sur nos observations cliniques nous avons émis l´hypothèse selon laquelle une méconnaissance de la maladie et une interprétation des symptômes comme étant intentionnels jugés négativement par les proches pourraient jouer un rôle dans le déclenchement d´une attitude critique, hostile et ou le surinvestissement viserait à corriger le dysfonctionnement du malade. Notre travail ne retrouve pas de lien significatif, en se basant sur des questions directes, avec aucune des dimensions explorées. En effet, il est probable que des questions directes ne reflètent pas les dispositions mentales et les véritables croyances des proches. Une alternative à cette interprétation est que les croyances et les représentations mentales peuvent agir indirectement par l´intermédiaire d´émotions négatives qu´elles pourraient provoquer à savoir la honte ou la colère. Leff et Vaughn [5] ont fait valoir une caractéristique importante chez les sujets avec un faible EE qui était la compréhension relativement rationnelle des problèmes du patient et de la reconnaissance des symptômes comme étant des signes de maladie. Greenley [38] et Hooley [4] de manière indépendante ont suggéré que les attitudes haute niveau d´EE pourraient être conceptualisées comme reflétant les tentatives de contrôle visant la restauration ou le changement du comportement du patient. Ces idées ont conduit à tester des hypothèses sur les processus psychologiques impliqués dans l'EE. Greenley [38] a montré que les parents les plus anxieux et craintifs seraient susceptibles d'essayer de contrôler ou de changer le comportement du patient. Plus encore, les parents qui considèrent le patient comme malade seraient moins susceptibles de faire face en utilisant le contrôle social.

Enfin, notre travail souffre d’un certain nombre de limites méthodologiques notamment imposés par les contraintes de l´exercice de la psychiatrie dans notre pays. En premier c´est notre outil de mesure, malgré une bonne spécificité et sensibilité dans la mesure des commentaires critiques et de l´hostilité, il ne rend pas compte du niveau du surinvestissement qui constitue une composante essentielle de l´échelle *CFI* reconnue comme étant le Gold standard en matière d´EE. Le recours au *FAS* est expliqué par sa disponibilité et sa facilité d´usage. Deuxièmement, nous avons adopté une approche directe pour l´exploration des croyances et représentations mentales liées à la maladie et ce dans une optique de simplification de la communication avec les proches des malades étant donné que le niveau culturel de la population étudiée est globalement bas. En outre, notre travail ne prend pas en compte la dimension affective chez les proches dans sa globalité, en effet la dimension dépressive, ainsi que les troubles anxieux chez les proches peuvent jouer un rôle dans la dynamique qui détermine le niveau EE et des études ultérieures devraient combler ce manquement étant donné que durant l´étude plusieurs proches ont exprimé une souffrance psychique ou ont montré des réactions émotionnelles à l´évocation de la relation avec le patient.

## Conclusion

La prise en charge de la schizophrénie néglige souvent les aspects psycho-sociaux qui revêtent un intérêt majeur et déterminent fortement le cours de la maladie. Si la psychoéducation prend de plus en plus de place dans cette prise en charge, il existe un besoin de compréhension des mécanismes psycho-sociaux qui interviennent à ce niveau et ce tenant compte des particularités culturelles locales. Nos résultats vont dans le sens que le milieu familial devient un facteur de rechute quand le poids de la charge portée par la famille est grand, quand le patient est consommateur à fortiori dépendant au cannabis et quand les membres de la famille ressentent de la honte vis-à-vis du malade. Tous ces éléments doivent être confirmés par d´autres études et doivent guider toute intervention de psychothérapie ou psychoéducation centrée sur la famille.

### Etat des connaissances sur le sujet

- La notion d´émotion exprimée désigne l´attitude des proches et le climat émotionnel dans lequel vit un patient ;
- Cette notion est associée à plus de rechutes et donc à une évolution plus défavorable de la maladie ;
- Ces résultats ont été reproduits dans plusieurs cultures.

### Contribution de notre étude à la connaissance

- Notre étude apporte les toutes premières données sur le niveau d´émotion exprimée au Maroc;
- Le niveau d´émotion exprimée dans notre contexte est associé à la consommation de cannabis et au sentiment de honte des proches.

## Remerciements

Nous remercions Dr Chouhani Wafae et Dr Chouhani Basmat Amal. Nous remercions également tous les patients et les familles ayant accepté de participer à l´étude. Ainsi que toute l´équipe médicale et paramédicale du service de psychiatrie. Nous remercions également le département d´épidémiologie de la Faculté de médecine de Fès.
